# Synthesis of copaiba (*Copaifera officinalis*) oil nanoemulsion and the potential against Zika virus: An *in vitro* study

**DOI:** 10.1371/journal.pone.0283817

**Published:** 2023-09-07

**Authors:** Tamara Carvalho, Marcela Guimarães Landim, Maria Letícia Duarte Lima, Cíntia Bittar, Beatriz Carvalho de Araújo Oliveira Faria, Paula Rahal, Milena Campelo Freitas de Lima, Valdir Florêncio da Veiga Junior, Graziella Anselmo Joanitti, Marilia Freitas Calmon

**Affiliations:** 1 Department of Biology, São Paulo State University—UNESP, Rua Cristóvão Colombo, São José do Rio Preto, Brazil; 2 Laboratory of Bioactive Compounds and Nanobiotechnology (LBCNano), University of Brasilia, Campus Universitário – Centro Metropolitano, Ceilândia Sul, Brasília, Federal District, Brazil; 3 Post-Graduation Program in Nanoscience and Nanobiotechnology, Institute of Biological Sciences, University of Brasilia, Campus Universitário Darcy Ribeiro, Brasília, Brazil; 4 Chemistry Section, Military Institute of Engineering, Praça Gen. Tibúrcio, Praia Vermelha, Rio de Janeiro, Brazil; Vietnam Academy of Science and Technology, VIET NAM

## Abstract

Zika virus (ZIKV) has spread all over the world since its major outbreak in 2015. This infection has been recognized as a major global health issue due to the neurological complications related to ZIKV infection, such as Guillain–Barré Syndrome and Zika virus Congenital Syndrome. Currently, there are no vaccines or specific treatments for ZIKV infection, which makes the development of specific therapies for its treatment very important. Several studies have been developed to analyze the potential of compounds against ZIKV, with the aim of finding new promising treatments. Herein, we evaluate the ability of a copaiba (*Copaifera officinalis*) oil nanoemulsion (CNE) to inhibit ZIKV. First, the highest non-cytotoxic concentration of 180 μg/mL was chosen since this concentration maintains 80% cell viability up to 96h after treatment with CNE in VERO cells resulted from MTT assay. The intracellular uptake assay was performed, and confirmed the internalization of the nanoemulsion in cells at all times analyzed. VERO cells were infected with ZIKV and simultaneously treated with CNE and the nanoformulation without oil (ENE) at the highest non-toxic concentration. The results evaluated by plaque assay revealed a viral inhibition of 80% for CNE and 70% for ENE. A dose-dependence assay revealed that the CNE treatment demonstrated a dose-dependent response in the viral RNA levels, whereas all ENE tested concentrations exhibited a similar degree of reduction. Taken together, our results suggest CNE as a promising nano-sized platform to be further studied for antiviral treatments.

## Introduction

The Zika virus (ZIKV), an arbovirus, belonging to the genus *Flavivirus*, has as its main vectors mosquitoes from the *Aedes* genus, and was first isolated in 1947 in the Zika forest, in Uganda [1, 2]. The worldwide propagation of its vectors, *Aedes aegypti* and *Aedes albopictus*, due to urbanization and deforestation processes, has resulted in the rapid spread of this arbovirus [3, 4]. The first outbreak of ZIKV occurred only in 2007 on Yap Island in the Federated States of Micronesia, where symptoms of fever, rash, joint pain, and conjunctivitis were observed [5, 6]. Since the emergence of ZIKV in Brazil in 2015, it has spread all over the Americas and has become a major global health issue due to the neurological complications related to ZIKV infection, such as Guillain–Barré Syndrome and Zika virus Congenital Syndrome [7–10].

Currently, there are no vaccines or specific treatments for ZIKV infection, which makes the development of specific therapies important [11, 12]. Several studies have analyzed bioactive compounds against Zika virus, including some specific targets, such as entry inhibitors, like Nanchangmycin, which is a natural compound that showed action in this step against ZIKV [13, 14]. In addition, some replication inhibitors, such as some nucleoside analogues, demonstrated an action against the RNA-dependent RNA polymerase of this virus [13]. Other studies analyzed the action of compounds targeting host proteins, like caspase inhibitors [15] and protein metabolism inhibitors [12, 16]. Thus, several studies have investigated bioactive compounds, such as those found in copaiba oil, obtained from the trunk of various *Copaifera* species [17]. In Brazil, there are more than twenty species of *Copaifera*, among the most abundants, we have the *C*. *officinalis* [18]. The major components of the oleoresin from *Copaifera officinalis*, used in the present study, are β-caryophyllene, allo-aromadendrene, germacrene B, β-bisabolene, δ-cadinene, and α-cadinene [17, 19, 20]. These secondary metabolites belong to the class of sesquiterpenes and several biological activities of this class have already been demonstrated in the literature, such as pro-oxidant, antioxidant, anti-inflammatory, antitumor, antiparasitic effect, etc [21].

The Copaiba oil has already shown antimicrobial activities, including an antiviral action [22–24]. These antimicrobial effects described in the literature for copaiba oil are related to the lipophilic characteristics of this oil, which may cause changes in the cell membrane, especially in relation to the antibacterial activities described in previous studies [24]. Besides that, one study reported the use of copaiba oleoresin aimed at the control of *Aedes aegypti*, which is the Zika virus vector. The authors demonstrated the potential of monoliths with this oil as a larvicidal [25]. Another study showed the use of plant essential oils, including copaiba oil, as a mosquito repellent, with action against *Aedes aegypti* [26]. In relation to antiviral activity against *Flavivirus*, the use of this oil has not yet been explored, which demonstrates the importance of the present work, which aimed to elucidate the potential of this oil against Zika virus.

Due to the limitations presented by hydrophobic active compounds, such as low solubility and stability, several studies associated these drugs with nanoemulsions. This association increases the effectiveness of treatment, leading to several benefits, such as improved compound delivery, a reduction in adverse drug effects, a reduction in therapeutic doses, increased bioavailability, and stability in biological fluids, among other effects [27–29]. Several studies in the literature have investigated the antiviral potential of nanoparticles or nanoemulsions [30] with inhibitory action against Zika and Dengue [31, 32]. Some activities have already been described for these nanoemulsion and copaiba oil systems, as for example, antimicrobial actions of the nanoemulsions associated with copaiba oil against the fungi *Paracoccidioides sp* [33], and the bacteria of the genus *Staphylococcus* [34].

In the current study, we used nanotechnology strategies to formulate a copaiba oil-based nanoemulsion (CNE) and evaluate the action of this nanosystem on cells infected with Zika virus. Our data show that both the structure of the nanoemulsion and its association with the oil demonstrate antiviral activity.

## Materials and methods

### Copaiba oleoresin chemical composition

The copaiba oleoresin was purchased from the Brazilian company FERQUIMA (São Paulo, Brazil), described as extracted from the species *Copaifera officinalis*.

The chemical composition of the copaiba oil was determined using gas chromatography coupled with mass spectrometry (GC-MS—Trace GC Ultra-ISQ Single Quadrupole, Thermo Scientific, Electron Impact, 70 eV, range from 40–400 u.m.a.). The oil was derivatized using trimethylsilyldiazomethane (TMSD; Sigma-Aldrich, Missouri, USA) in order to transform the diterpenic carboxylic acids into their correspondent methyl esters. This modification is important to enable the analysis by GC, since carboxylic acids do not present good chromatographic resolution. With the retention times of all the terpenes, Retention Indices (RI, from Van der Dool-Kratz equation) were obtained by comparison with a hydrocarbon linear homologous series and correlated with data from the literature [35] for sesquiterpenes, and data from our databases for previously isolated and characterized diterpenes. Similarly, mass spectra were compared with the research group library spectra, for diterpenes, and the NIST spectra library, for sesquiterpenes. The derivatized copaiba oil (1 μL, split 1:20, Injector temperature 250 °C) was inserted in a capillary column DB5/MS (30 m x 0.25 mm ID x 0.25 μm) using Helium (2 mL/min) as carrier gas. The oven program was set to initiate at 90 to 160 °C (2.5 °C/min), and then from 160 to 290 °C (8.0 °C/min), with a 5 minute final isotherm.

### Development and characterization of copaiba oil nanoemulsion (CNE)

I) Formulation development formulation development

The surfactant used in the nanoemulsion formulation was LIPOID E 80 ^™^ egg lecithin (Steinhausen, *Switzerland*) which comes from a natural source and is considered safe by the US Food and Drug Administration [36]. It is composed mostly of the phospholipid phosphatidylcholine (80%).

The development of the nanoemulsion was based on a previously described method. First, the surfactant and oil masses were measured (180 and 90 mg respectively) respecting the 2:1 (w/w) ratio of surfactant to oil. Then, 5 mL of deionized water was added and the mixture was immediately subjected to sonication at 20 kHz under an ice bath for 6 minutes (Vibra-Cell ^™^, Sonics & Materials Inc., USA) to form the concentrated nanoemulsion. Subsequently, a 1:12.5 (v/v) dilution of this concentrate was again subjected to sonication as described above, resulting in a final concentration of 1.44 mg/mL of oil. To test whether the vehicle itself has any biological activity, formulations were made without oil (ENE), containing only the surfactant and water, in the same proportions as the respective nanoemulsion.

In parallel, to obtain fluorescent nanoemulsions, aluminum-chloride phthalocyanine (AlClPc) (Sigma-Aldrich, Missouri, USA) was added to the nanoemulsion in the step of weighting the components before sonication, reaching a final concentration of 0.04 mg/mL.

The developed formulations were stored at 4°C, under dark conditions, until further analysis.

II) Analysis using ZetaSizer

The formulations were characterized according to their physicochemical parameters: hydrodynamic diameter [35], polydispersity index (PDI), and Zeta potential (ZP) using the ZetaSizer^®^ Nano ZS90 (Malvern, UK) at room temperature (25°C) and with detection of light scattering at an angle of 90°. First, the nanoemulsions were diluted in 1:10 (v/v)– 100 μL of nanoemulsion + 900 μL of water (deionized ultra-pure water). After that, 1 mL of each sample was added in a polystyrene cuvette, and then submitted for a ZetaSizer reading (Malvern, UK). To calculate the Z-average, three readings of each sample were obtained. The pH of the samples was measured with a pH indicator stripe prior to each zeta potential analysis [37]. The physicochemical characterization was performed in three independent experiments. For stability analysis, formulations were stored at 4°C under dark condition and their physicochemical parameters were analyzed after 1, 7, 15, 30, and 60 days, as described above. This approach is in accordance with Singh et al (2017) and Sánchez-López et al (2019) [38, 39].

III) Scanning Electron Microscopy (SEM)

The morphological characterization of CNE was performed by Scanning Electron Microscopy (SEM) using JSM-7001F microscope (Jeol, Japan). The Samples were left to dry at room temperature (24 h), and then contrasted with 2% OsO_4_ vapor for 20 min. For scanning electron microscopy (SEM), samples were diluted 1:300 (v/v) in distilled water, and deposited on a smooth carbon tape adhered to a stub surface, contrasted with 2% OsO4 vapor for 30 min, and left to dry at room temperature (24 h). After complete droplet drying, the samples were metallized with gold in a Sputter Coater (Leica, EM SCD 500, Austria).

VI) Transmission electron microscopy (TEM)

The morphological characterization of CNE was also performed by Transmission electron microscopy (TEM) by using JEM-1011 (Jeol, Japan) microscope. Samples were diluted 1:300 (v/v) in distilled water, and deposited on a 200 mesh copper grid covered with a Formvar ultra film. The samples were left to dry at room temperature (24 h), and then contrasted with 2% OsO_4_ vapor for 20 min.

### Cells and virus

VERO E6 cell lines from African green monkey kidney (*Cercopithecus aethiops*) (ATCC CRL-1586) and *Aedes albopictus* C6/36 (ATCC CRL-1660) cells were used. VERO E6 cells were cultured in Dulbecco’s modified Eagle medium (DMEM) (GIBCO-BRL, Life Technologies, California, USA) supplemented with 10% fetal bovine serum (Cultilab, São Paulo, Brazil), 100 IU/mL penicillin, 100 μg/mL streptomycin (P/S), and 1% (v/v) non-essential amino acids (GIBCO-BRL, Life Technologies, California, USA) and maintained at 37°C in a humidified 5% CO_2_ incubator. *Aedes albopictus* cell line C6/36 was grown in Leibovitz L-15 (L-15) medium (Cultilab, São Paulo, Brazil), with the same supplementation as the other cell line, maintained at 28ºC and without CO_2_ enrichment incubation.

The *Aedes albopictus* C6/36 cell line was used for infection and propagation of the Zika virus used in this work.

The Brazilian strain of Zika virus (ZIKV^BR^) used in the experiments was isolated from a feverish case in Paraíba state, in northeastern Brazil, and was kindly donated by Dr. Pedro Vasconcelos, Instituto Evandro Chagas [40]. The access numbers deposited at Genbank for the ZIKV genome are: KU321639, KU365777 to KU365780, KU729217, and KU729218.

### Analysis of nanoemulsion cytotoxicity

The cytotoxicity profile of the formulation without oil (ENE), nanoemulsion containing copaiba oil (CNE), and free copaiba oil (FCO) were measured by the MTT [3-(4,5-dimethylthiazol- 2-yl)-2,5-diphenyl tetrazolium bromide] method (Sigma-Aldrich, Missouri, USA). This experiment was performed in three independent events.

Twenty-four hours before treatment, 5x10^3^ VERO E6 cells were plated in a 96-multi-well plate. On the following day, CNE and FCO were added at concentrations ranging from 5.6, 11.2, 22.5, 45, 90, 180, and 360 μg/mL. Since the tested concentrations were based on copaiba oil, the ENE treatment was performed with the same volume used for each concentration of CNE. Cells were incubated for 24, 48, and 96 hours for VERO E6, in triplicate. After these times, the culture medium was removed from each well and exchanged for MTT solution (1 mg / mL in 100 μL of culture medium without supplementation) (Sigma-Aldrich, Missouri, USA). Cells were incubated for 30 minutes at 37°C. After the formation of the formazan crystals, the MTT solution was removed and 100 μL of DMSO (Synth, Sao Paulo, Brazil) were added per well. The absorbance was then measured at 572 nm, using a plate spectrophotometer (FLUOstar Omega/BMG LABTECH, Offenburg, BW, DE). The maximum non-cytotoxic concentration was determined considering a cut-off of 80% of cell viability.

### Intracellular uptake

Approximately 10^5^ VERO E6 cells were plated in 6-well plates, and after 24 hours for complete cell adherence, the cells were incubated with nanoemulsion at the concentration previously established by MTT. For this assay, the nanoemulsion was labeled with the molecule aluminum-chloro-phthalocyanine (AlClPc), which has an excitation wavelength of 350 nm and an emission peak of 680 nm (range between 650–700 nm), to observe the fluorescence. After 1, 2, 3, and 48 hours of incubation with the nanoemulsion, the medium was removed, and the cells were washed with PBS, before being viewed and photographed using a fluorescence microscope (Zeiss Axio Vert. A1).

### Viral inhibition assay

To perform the ZIKV inhibition assay, initially 2x10^5^ VERO E6 cells were plated per well in a 12-well plate. Treatment groups were then prepared: Group A (control) = ZIKV^BR^ + pure DMEM medium; Group B (CNE) = ZIKV^BR^ + nanoemulsion containing copaiba oil; Group C (ENE) = ZIKV^BR^ + formulation without oil. For this assay, the highest non-cytotoxic concentration was used. After 1 hour, fresh medium containing 1% FBS, 1% P/S, and 1% Carboxymethylcellulose (CMC) was added to Group A and nanoemulsions (empty and containing copaiba oil) were added to groups B and C at the proper concentrations. Cells were incubated at 37°C and 5% CO_2_ for 96 hours. After this time, cells were fixed with 10% formaldehyde for 30 min and stained with 1% crystal violet (Sigma-Aldrich, Missouri, USA) and the viral titer was quantified and compared. This experiment was performed in three independent events.

### Analysis of the dose dependence of nanoemulsions

For the dose dependence analysis of nanoemulsions, 10^5^ VERO E6 cells were plated in a 12-well plate. After 24 h, the cells were incubated with nanoemulsions CNE and ENE at 5.6, 11.2, 22.5, 45, 90, 180, and 360 μg/mL and 1.16x10^4^ PFU/mL of ZIKV^BR^. Cells were incubated for 1 hour at 37°C and 5% CO_2_. After 1 hour, complete medium was added to the cells along with the nanoemulsions at each concentration, except for the untreated control. Cells were incubated for 48 hours at 37°C and 5% CO_2_. The supernatant samples were collected from each well for subsequent RNA extraction and cDNA synthesis, and real time PCR was performed. This experiment was performed in three independent events.

### ZIKV RNA quantification real time PCR (qPCR)

In order to quantify the viral RNA, the qPCR assay was performed using *primers* ZIKV 1086, ZIKV 1162c, and probe 1107-FAM described in Lanciotti et al (2008) using the TaqMan^®^ Universal PCR Master Mix in AmpErase UNG (Applied Biosystems) according to the manufacturer’s instructions. Reactions were read in the QuantStudio 12K Flex Real Time PCR System (Applied Biosystems Inc/Life Technologies, California, USA) [41]. Quantification of ZIKV RNA copies was obtained by the standard curve method.

### Statistical analysis

For statistical analysis of the groups, comparisons were performed by analysis of variance (One-Way ANOVA) followed by the Tukey test. *P* values <0.05 were considered significant. Statistical analyses were performed using GraphPad Prism 5 software (GraphPad Software, Inc., CA, USA). Microsoft Excel was also used to aid in data processing.

## Results

### Analysis of copaiba oil chemical composition

The chemical composition observed showed that this oil is clearly a pure copaiba oil, full of sesquiterpenes, with low amounts of diterpenes, and no other constituent, such as fatty acids, considered common adulterations of copaiba oils (Table 1). Diterpenes, approximately 10% of the oil content, are mainly derivatives of copalic acid, the labdane diterpene that, together with caryophyllene, is considered a biomarker of copaiba oils.

**Table 1:** Chemical components of copaiba oil [Retention Indices (RI) related to their Retention Temperatures (RT)].

Peaks	Components	RI	Copaiba oil	
			RT	Area (%)
01	δ-elemene	1335	8.64	0.38
02	α- cubebene	1348	9.02	0.26
03	α-copaene	1374	9.87	2.80
04	β-elemene	1389	10.42	0.83
05	cyperene	1398	10.64	0.26
**06**	**β-caryophyllene**	1417	11.37	**59.89**
07	(E)-α-bergamotene	1432	11.97	3.22
**08**	α-humulene	**1452**	12.54	**10.38**
09	allo-aromadendrene	1458	12.81	0.38
10	germacrene D	1480	13.57	3.79
11	β-cis-guayene	1492	14.14	0.40
12	α-muurolene	1500	14.37	0.30
13	β-bisabolene	1505	14.76	0.71
14	δ-cadinene	1522	15.26	1.39
15	caryophyllenol	1570	16.90	0.71
**16**	caryophyllene oxide	**1582**	17.40	**1.75**
17	humulene epoxide II	1608	**-**	**-**
18	junenol	1618	**-**	**-**
19	10-epi-α-muurolol	1640	19.87	0.28
20	δ-cadinol	1644	20.05	0.18
21	α-cadinol	1652	20.36	0.31
**22**	copalic acid	**-**	36.38	**3.86**
23	cholavenic acid	**-**	36.96	0.14
25	hardwickic acid	**-**	37.81	0.16
26	pinifolic acid	**-**	38.13	0.19
27	agatic acid	**-**	39.01	2.00
28	3-hydroxy-copalic acid	**-**	39.14	0.51
**29**	3-acetoxy-copalic acid	**-**	40.09	**3.18**
Identified components (%):			**98.26**	

### Analysis of CNE colloidal stability

**Fig 1:**
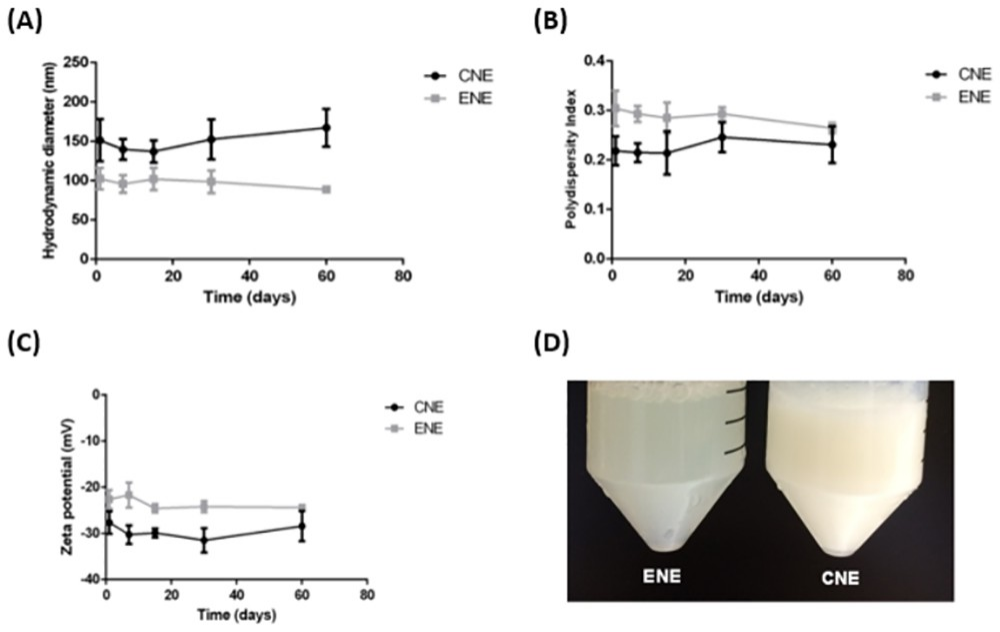
Colloidal stability parameters of copaiba oil nanoemulsion (CNE) and nanoformulation without oil (ENE) over time of storage (1, 7, 15, 30, and 60 days) at 4°C according to hydrodynamic diameter (A), polydispersity index (B), and zeta potential (C). Macroscopic aspect of CNE and ENE (D). (Data available: S1 and S2 Figs).

Considering that size, size distribution, and surface charge are factors which directly influence nanoemulsion stability [42], these parameters were measured over time and are shown in Fig 1. The colloidal stability of the CNE and ENE formulations was evaluated by observing the variation in the values of hydrodynamic diameter, polydispersity index (PDI), and Zeta potential (ZP) of five batches of nanoemulsions, during the period of up to 60 days after their preparation (Fig 1).

For the CNE, the hydrodynamic diameter (HD) average was 152.3 nm ± 15.1 nm, the PDI average was 0.230 ± 0.016, and the ZP average was -29.57 mV ± 1.93 mV (pH 6). For the ENE, the HD average was 118 nm ± 0.9 nm, the PDI average was 0.286 ± 0.010, and the ZP average was -23.6 mV± 0.8 mV (pH 7) (Fig 1). The CNE and ENE showed no signs of flocculation, creaming, sedimentation, or phase separation in their macroscopic aspect (Fig 1D).

Neither CNE nor ENE showed significant differences in HD, PDI, or ZP between the first day after formulation and the days 7, 15, 30, and 60 of the analysis, presenting colloidal stability during this time interval at 4°C (Fig 1).

Additionally, CNE nanoemulsion containing the fluorescent marker AlClPc was produced, and the physico-chemical properties were similar to the standard CNE (HD = 157.1 ± 2.5; PDI = 0.235 ± 0.011).

**Fig 2:**
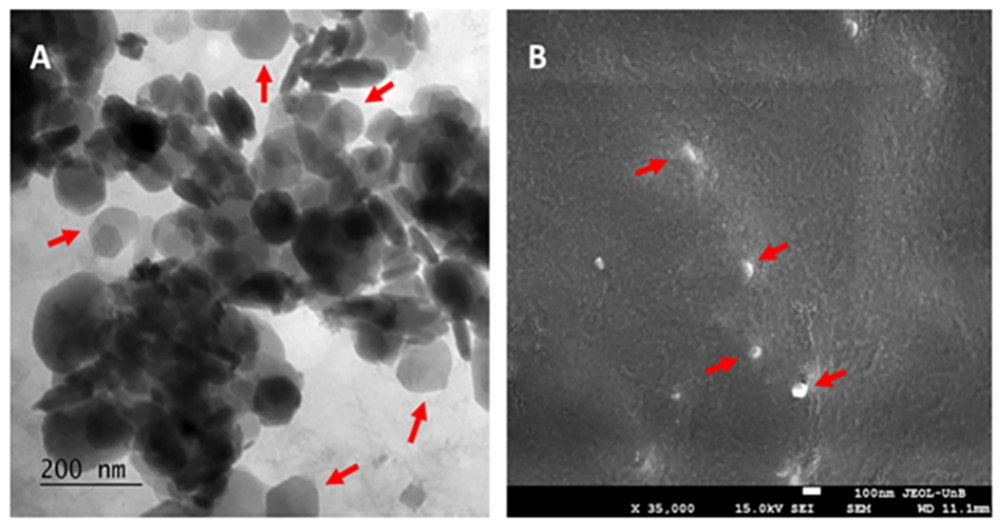
Representative ultramicrographs of copaiba oil nanoemulsion (CNE), acquired by transmission (A) and scanning (B) electron microscopy. Red arrows indicate CNE nanodroplets.

The CNE ultrastructural analyses were obtained by transmission (TEM) and scanning (SEM) electron microscopy. While size data obtained by dynamic light scattering (DLS -Zetasizer^®^) considers the hydrated particle size (hydrodynamic diameter), TEM and SEM provide its dry size, given the sample processing. The representative ultramicrographs (Fig 2), acquired by both techniques, show spherical or quasi-spherical morphologies for CNE nanodroplets.

### Analysis of nanoemulsion cytotoxicity

**Fig 3:**
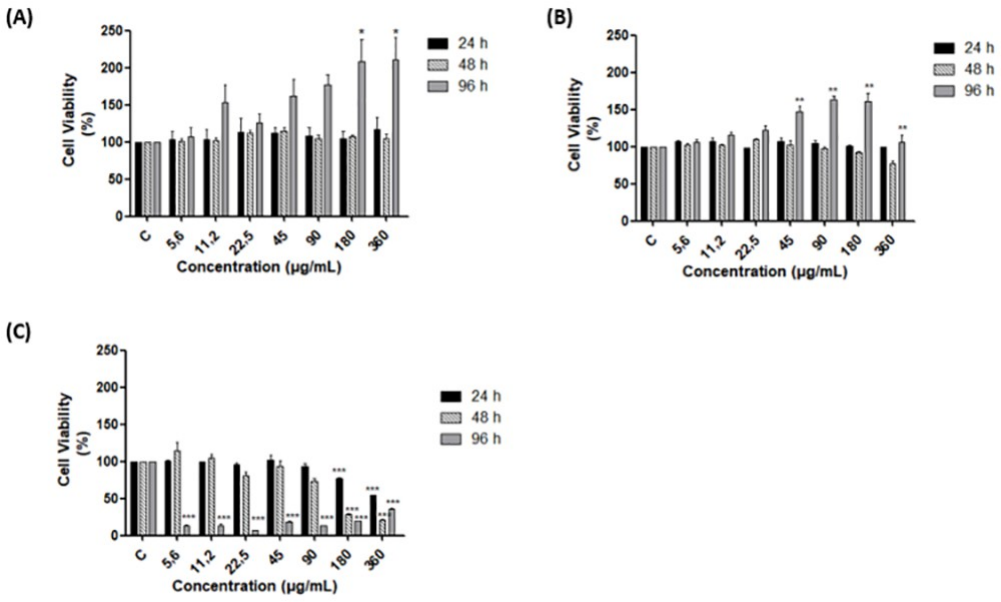
Cell viability of VERO E6 cells treated with nanoformulation without oil (ENE), copaiba oil nanoemulsion (CNE), and free copaiba oil (FCO), for 24, 48, and 96 hours in concentrations of 5.62; 11.25; 22.5; 45; 90; 180; and 360 (μg/mL). (A) Treatment with ENE. (B) Treatment with CNE. (C) Treatment with free copaiba oil. C: control cells. Representative of statistical significant difference between concentrations of treatments and the control (*: *p*<0.05; **: *p*<0.01; ***: *p*<0.001). (Data available: S3–S5 Figs).

To investigate CNE and ENE cytotoxicity, VERO E6 cell lines were treated with different concentrations (5.62; 11.25; 22.5; 45; 90; 180; and 360 μg/mL) of the formulations and free copaiba oil. Data were analyzed by MTT assay after 24, 48, and 96 h of incubation. Neither formulation was cytotoxic at any time evaluated, in any tested concentration (Fig 3A). However, free copaiba oil showed high levels of cytotoxicity after 48 hours (Fig 3C).

After statistical analysis, we were able to determine that when comparing the viability resulting from the treatment with each formulation, there were significant differences between ENE and the copaiba oil treatment for 24 hours (*p*<0.01) and for 48 hours (*p*<0.05). On the other hand, for the 96-hour treatment, both comparisons between the treatments ENE and copaiba oil (*p*<0.001), and CNE and copaiba oil (*p*<0.001) demonstrated statistical differences, demonstrating that this time period presents the most significantly different cell viability values resulting from the treatment with the nanoformulations and the copaiba oil.

For CNE for the 24-hour treatment, there were no statistical differences between any concentration and the control. However, for the 48-hour treatment, there was a statistically significant difference between the concentration of 360 μM and the control (*p*<0.01), while for the 96-hour treatment, there was a statistical difference between the concentrations 45, 90, and 180 μM and the control (*p*<0.01). For the antiviral testing we chose the 180 μM concentration because it is the highest one with viability above 80% for all times analyzed.

For ENE, in the periods of 24-hour and 48-hour of treatment, there were also no statistical differences between any concentration and the control. However, for the 96-hour treatment, there was a statistical difference for the concentrations of 180 and 360 μM (*p*<0.05).

Finally, for the copaiba oil treatment, it is possible to notice that in the first and second periods of treatment, there was a statistical difference for the concentrations of 180 and 360 μM (*p*<0.001), while for the 96-hour treatment, all the concentrations demonstrated a significant difference (*p*<0.001), as shown by the level of toxicity of all concentrations in this period of treatment.

Based on the MTT results, VERO E6 cells maintained a cell viability above 80% up to the concentration of 180 μg/mL at all times analyzed, for the treatment with ENE and CNE. It is also important to highlight that, as can be seen from the graphs, treatment with free copaiba oil showed expressive cytotoxicity (Fig 3C), which made it impossible to analyze the action of the free oil on ZIKV inhibition in later experiments.

### Intracellular uptake

After performing cytotoxicity analysis of nanoemulsions in VERO E6 cells, the next step was to confirm the cellular uptake of nanoemulsions at 1, 2, 3, and 48 hours of incubation. As copaiba oil is not fluorescent, aluminum chloro-phthalocyanine (AlClPc) was used for labeling the CNE, without showing significant alterations in the physicochemical properties of this nanoemulsion.

**Fig 4:**
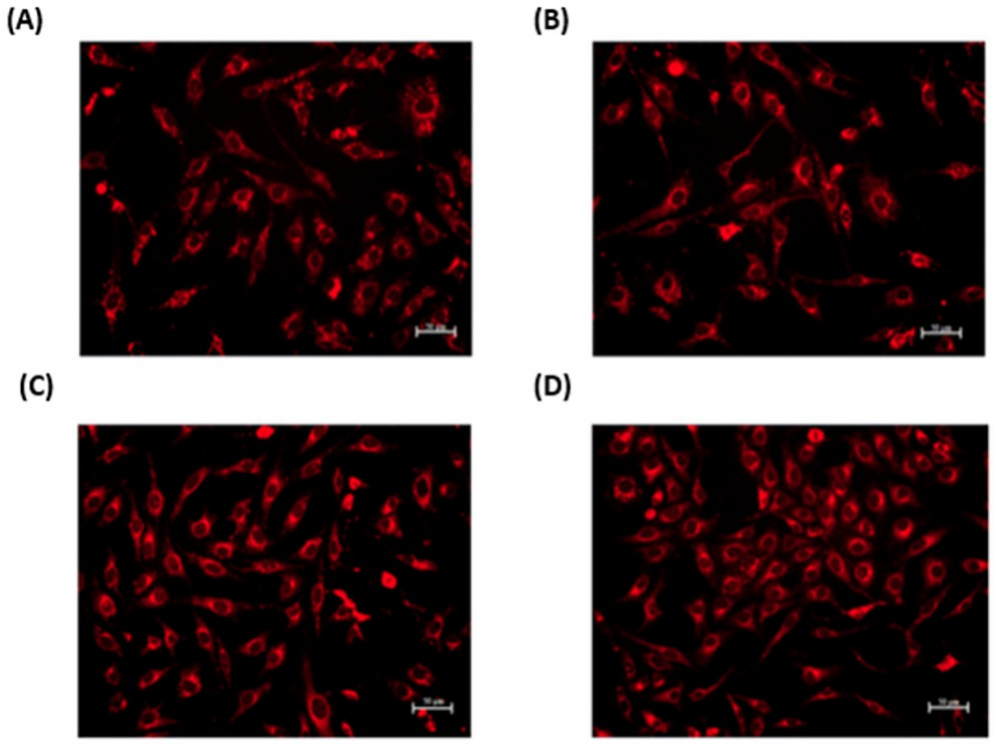
Fluorescent microscopy images of cellular uptake of copaiba oil nanoemulsion—CNE (180 μg/mL) in VERO E6 after different periods of incubation. The pictures were taken (A) 1 hour (B) 2 hours, (C) 3 hours, and (D) 48 hours after nanoemulsion incubation. Scale bar: 50 μm.

In VERO E6 cells, the nanoemulsion internalization can be observed at all times analyzed, especially in the cytoplasm of the cells (Fig 4).

### Viral inhibition assay

**Fig 5:**
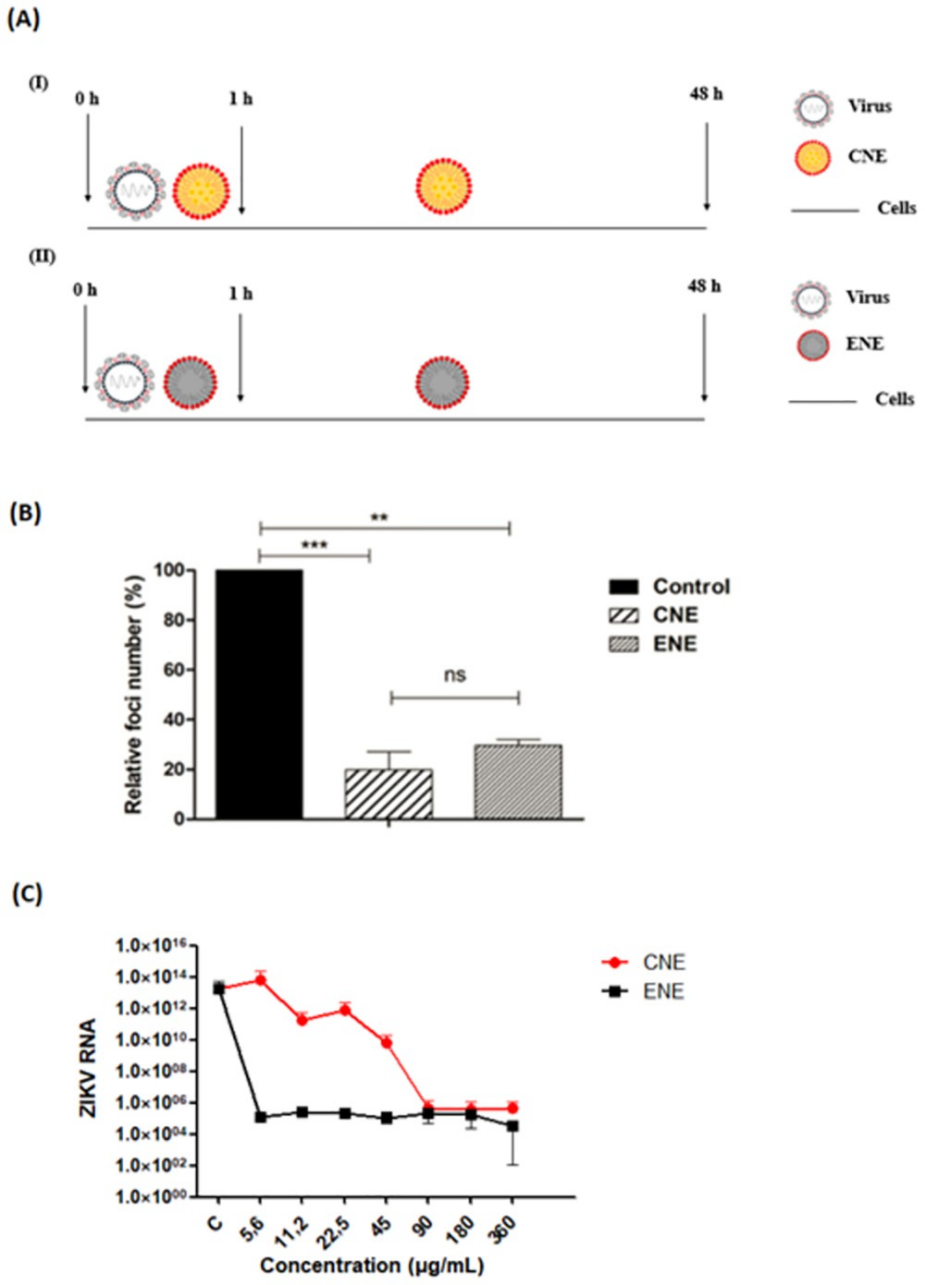
VERO E6 cells incubated with nanoformulations presented viral activity inhibition. (A) Representative scheme of the viral inhibition assay and the dose-dependence assay. First (I), representing the test performed with the copaiba oil nanoemulsion (CNE), and, second (II), representing the test performed with the nanoformulation without oil (ENE). (B) Relative foci number (%) resulting from the viral inhibition assay in VERO E6 cells infected with ZIKV. These assays were performed in two independent events. Both treatments showed statistical difference in relation to control cells (**: p <0.01 and ***: p <0.001). (C) ZIKV RNA resulting from the dose dependence of CNE and ENE in VERO E6 cells infected with ZIKV. (Data available: S6 and S7 Figs).

Firstly, to analyze the possible ZIKV inhibition effect of the formulations, the viral inhibition assay was perfomed. VERO E6 cells treated with ENE and CNE showed 70% and 80% of viral inhibition activity, respectively, when compared to control cells (*p* <0.001 and *p* <0.01, respectively) (Fig 5B).

### Analysis of the nanoemulsions dose-dependence

In order to analyze the dose-dependence of ENE and CNE, a dose dependence assay was performed. VERO E6 cells incubated with CNE demonstrated a dose-dependent response in viral RNA levels. The ENE showed a decrease in viral RNA in all volumes tested (Fig 5C).

## Discussion

Due to the current absence of vaccines and approved treatments against ZIKV infection, studies analyzing the action of antiviral compounds for this virus are of great importance [43]. Based on this and the various biological activities already proposed in the literature for copaiba oil, along with the use of nanoemulsions to potentiate the antimicrobial activities of natural oils, the present work studied the action of nanoemulsions containing copaiba oil as a possible antiviral tool against ZIKV [44, 45].

The chemical composition of copaiba oil is an oil-resin with volatile liquid sesquiterpenes dissolved in a resinoid diterpenic acid mixture. Some oils present a huge amount of sesquiterpenes, compared to the quantity of diterpenes, such as *C*. *officinalis* L. and, mainly, *C*. *multijuga* Hayne, corroborating our results about the composition of the copaiba oil used in the present work (Table 1) [46–48]. Regarding to the nanoemulsion structure used herein, it is composed mainly of water, surfactant (egg lecithin), and copaiba oil. This formulation presents amphiphilic characteristics, showing a hydrophilic surface and a hydrophobic inner compartment able to carry the bioactive compounds found in copaiba oil.

Mason et al (2006) and McClements et al (2012) agree that nanoemulsions are kinetically stable dispersions characterized by presenting nanodroplets with an average diameter inferior to 200 nm. In the present work, both formulations presented a hydrodynamic diameter lower than 200 nm, therefore in agreement with the definition of nanoemulsions stated in the literature [49, 50]. In addition, PDI values of the formulations were inferior to 0.3, indicating that they are in the value range closest to that considered homogeneous within the PDI scale according to Bhattacharjee (2016) [51]. The ZP of the formulations was negative, as also found in a hydrogel containing nanoemulsified copaiba oil used for anti-inflammatory treatments [42, 52].

Morphological analysis by TEM and SEM showed CNE with a spherical or quasi-spherical shape and nanodroplet diameters of approximately 100 nm (Fig 2). This diameter value is different from data obtained by dynamic light scattering (DLS -Zetasizer^®^) since microscopic techniques analyze the dry size while DLS data consider hydrated particle size [37].

It is important to highlight that nanoemulsion stability is crucial for efficient delivery and protection of the encapsulated compounds after production [42, 53]. Interestingly, the present data emphasize the 60-days stability profile of CNE and ENE since both formulations remained stable when stored at 4°C over the 60 days of analysis, considering the parameters of hydrodynamic diameter, PDI and ZP. Some studies considered nanoemulsions evaluated for 42 days [54] and 56 days [55] as long-stable, corroborating our period of analysis of nanoemulsion stability.

Nanoemulsions are systems that present the major advantage of a reduction in drug toxicity. In the cell viability assay, free copaiba oil was analyzed and showed expressive cytotoxicity in the VERO E6 cell line. In contrast, no cytotoxicity was observed after CNE and ENE treatments. These results could possibly be due to different internalization routes and intracellular targets promoted by the nanoencapsulation of the oil, which could prevent cell damage [56, 57]. Interestingly, we notice an increase in the cell viability of VERO E6 cells treated with higher concentrations of ENE during 96 hours. This could be due the composition of the nanoformulation, mainly constituted of phosphatidylcholine (~80%), a major component of membranes, source for lipid second messengers and determinant of cell cycle progression [24]. Corroborating our results Gandola et al., (2014) observed that high concentration of phosphatidylcholine nanoparticles significantly increased cell viability of MCF-7 cells [58, 59]. We do not observe similar behavior in VERO E6 cells treated with CNE probably due to the presence of the copaiba oil.

After analyzing cell viability, it was possible to confirm that the intracellular uptake of CNE was efficient at all times and in the cell line analyzed. Due to the different morphologies and characteristics (*e*.*g*. size, surface charge) that nanoparticles can have, they can be internalized in cells in different ways [60]. In addition, the nanoparticle location inside the cells is of great importance to confirm whether they are absorbed by the cells and their possible mechanisms of action [61]. In the present study, it was observed that the CNE nanodroplets are present mainly in the cytoplasm of VERO E6 cells. This internalization of the nanoemulsion in the cell cytoplasm may be related to the mode of action of the NE structure, since for this assay only ENE with AlClPc markers were used. The study by Jiang et al (2013) also used a lecithin-based nanoemulsion and demonstrated a time-dependent intracellular uptake, observing an increase in fluorescence intensity according to the time of exposure to the compound, which corroborated that observed in the present study [62].

This cytoplasmic localization could also be related to efficient delivery and may trigger a better treatment [63]. Nanoformulation internalizations that have cytoplasmic targets can result in improvement in the efficiency of the treatment, as the ZIKV replication cycle occurs in the cytoplasm [64]. Thus, for the present work, we can hypothesize that the nanoformulation action against Zika virus could be related to inhibition of steps post-entry of the virus in the cell.

The initial experiment to analyze the possible potential of the nanoformulations in inhibiting ZIKV at a maximum non-toxic concentration of 180 μg/mL at any stage of its cycle demonstrated a reduction in relative foci numbers of approximately 70% and 80% for both ENE and CNE, respectively (Fig 5B). A Subsequent assay revealed that ZIKV inhibition by CNE is dose-dependent (Fig 5C). Other studies demonstrated dose-dependent inhibition of natural compounds in ZIKV. Mounce et al (2017) discussed the effects of curcumin in a dose-dependent reduction on the infectivity of the enveloped viruses Chikungunya, Zika, and Vesicular Stomatitis virus in HeLa cells and related these results to curcumin structure [65]. In addition, the study of Behrendt et al (2017), using the compound Pentagalloylglucose, showed extracellular inhibition in a dose dependent manner, using Vero B4 cells [66]. Furthermore, some studies showed a dose-dependent inhibition of a stage of the ZIKV cycle by the compounds Suramin and Floxuridine, demonstrating an effect on the replication step [67, 68].

Since ENE (formulation without oil) also has antiviral activity, part of the antiviral effect of CNE might be related to the composition of egg lecithin (mainly phosphatidylcholine) and its direct effect on the viral envelope, that has a lipoprotein composition, and/or on the host cells. Studies demonstrated the inhibitory activity of a lipid nanoemulsion derived from natural food components on the flavivirus ZIKV and DENV [32, 69]. In addition, the bactericidal action of copaiba oil on Gram positive strains with a cell wall rich in peptidoglycan, could be related to a possible action of the copaiba oil on ZIKV viral glycoproteins and induction of morphological alterations in its integrity [45, 70, 71]. Drugs with this kind of effect are interesting because they act on the viral particle before it enters the cell, preventing cell damage from infected tissue.

The effects of CNE uptake in host cells might also play a role in the antiviral mechanism. It is known that viruses, specially *Flavivirus*, depend on lipids to succeed in several crucial steps during their life cycle and that targeting the cellular lipid metabolism has been explored as an antiviral strategy [72–74]. Considering that exogenous metabolites, including lipids, are potent modulators of cell function [75], it is possible that the exogenous intake of phosphatidylcholine, due to nanodroplet cell internalization, might modulate cell lipid metabolism, resulting in impairment of the virus life cycle.

To the best of our knowledge, this is the first study to report the physicochemical stability and the antiviral effect of copaiba oil-based nanoemulsion (CNE) against ZIKV. It is important to emphasize that the nanoformulations developed herein were obtained through low-cost, and eco-friendly synthesis approaches. Although the precise mechanism of action of CNE remains to be elucidated, the present data highlight the potential of CNE as an antiviral tool against ZIKV infection and open the perspective for similar actions in other enveloped viruses. Detailed investigations of the action of CNE in each step of ZIKV infection are underway.

## Conclusions

In conclusion, it was found that the nanoemulsion containing copaiba oil presents antiviral potential against ZIKV and was stable over the 60 days of analysis considering the parameters analyzed. The data indicate that both the structure of the nanoemulsion and its association with the oil present antiviral activity. The nanoformulations showed low cytotoxicity and antiviral activity against ZIKV. Although the results obtained until now evidence the potential of both nanoformulations as a novel therapeutic approach to inhibit ZIKV, further studies are needed to confirm the mechanism of action that these systems use to produce these effects.

## Supporting information

S1 Fig (PDF)

S2 Fig (PDF)

S3 Fig (PDF)

S4 Fig (PDF)

S5 Fig (PDF)

S6 Fig (PDF)

S7 Fig (PDF)
