# Supplementary material for: Synthesis of copaiba (Copaifera officinalis) oil nanoemulsion and the potential against Zika virus: An in vitro study
Source: PLoS One. 2023 Sep 7;18(9):e0283817. doi: 10.1371/journal.pone.0283817 (PMC10484457; doi:10.1371/journal.pone.0283817)
Supplement: S1 Fig — (PDF) [file pone.0283817.s001.pdf]

S1 Table: Partial data used for the construction of the graphs for the Figure 1 (A, B, and C).

Type	Sample Name	Temperature °C	Hydrodynamic diamenter d.nm	Polydispersity Index	Zeta potential mV
Size	ENE 1 D1	25	91,32	0,272	
Size	ENE 1 D2	25	89,35	0,333	
Size	ENE 1 D3	25	89,93	0,346	
Zeta	ENE 1 D1	25			-19,7
Zeta	ENE 1 D2	25			-18,1
Zeta	ENE 1 D3	25			-21,2
Size	CNE 1 D 1	25	142,8	0,222	
Size	CNE 1 D 2	25,1	138,9	0,214	
Size	CNE 1 D 3	25	144,9	0,220	
Zeta	CNE 1 D 1	25			-24,5
Zeta	CNE 1 D 2	25			-25,2
Zeta	CNE 1 D 3	25			-24,5
Size	CNE 7 D 1	25,0	144,7	0,216	
Size	CNE 7 D 2	25,0	142,5	0,198	
Size	CNE 7 D 3	24,9	142,1	0,224	
Zeta	CNE 7 D 1	25,0			-30,5
Zeta	CNE 7 D 2	25,0			-29,4
Zeta	CNE 7 D 3	25,0			-30,5
Size	ENE 7 D 1	24,9	90,85	0,289	
Size	ENE 7 D 2	25,0	89,52	0,286	
Size	ENE 7 D 3	25,0	88,84	0,300	
Zeta	ENE 7 D 1	25,0			-21,2
Zeta	ENE 7 D 2	25,0			-17
Zeta	ENE 7 D 3	25,0			-18,1
Size	CNE15 D1	25,0	148,4	0,199	
Size	CNE15 D2	25,0	145,6	0,190	
Size	CNE15 D3	25,0	146,1	0,197	
Zeta	CNE15 D1	25,0			-32,1
Zeta	CNE15 D2	25,0			-31,3
Zeta	CNE15 D3	25,0			-30,1
Size	ENE15 D1	25,0	91,1	0,333	
Size	ENE15 D2	25,0	92,36	0,271	
Size	ENE15 D3	25,0	90,37	0,304	
Zeta	ENE15 D1	25,0			-21,8
Zeta	ENE15 D2	25,0			-27
Zeta	ENE15 D3	25,0			-23,9
Size	CNE30 D1	25,0	148	0,195	
Size	CNE30 D2	25,0	142,7	0,227	
Size	CNE30 D3	25,0	144,3	0,210	
Zeta	CNE30 D1	25,0			-35
Zeta	CNE30 D2	25,1			-35,3
Zeta	CNE30 D3	25,1			-33,2
Size	ENE30 D1	25,0	90,86	0,283	
Size	ENE30 D2	25,0	89,51	0,277	
Size	ENE30 D3	25,0	90,39	0,307	

S1 Table: Partial data used for the construction of the graphs for the Figure 1 (A, B, and C).

Zeta	ENE30 D1	25,0			-21,4
Zeta	ENE30 D2	25,0			-25,6
Zeta	ENE30 D3	25,0			-22,9
Size	CNE60 D1	25,0	152,3	0,182	
Size	CNE60 D2	25,0	149,2	0,228	
Size	CNE60 D3	25,0	149,7	0,204	
Zeta	CNE60 D1	25,0			-32,2
Zeta	CNE60 D2	25,0			-30,2
Zeta	CNE60 D3	25,0			-29,8
Size	ENE60 D1	25,0	87,75	0,271	
Size	ENE60 D2	25,0	87,31	0,274	
Size	ENE60 D3	25,0	91,03	0,271	
Zeta	ENE60 D1	25,0			-23,9
Zeta	ENE60 D2	25,0			-27,6
Zeta	ENE60 D3	25,0			-21,6

(ENE 1 D 1 = Empty nanoemulsion, one day after synthesis, replicate 1 / CNE 1 D 1 = Copaiba nanoemulsion, one day after synthesis, replicate 1).
