# Supplementary material for: Synthesis of copaiba (Copaifera officinalis) oil nanoemulsion and the potential against Zika virus: An in vitro study
Source: PLoS One. 2023 Sep 7;18(9):e0283817. doi: 10.1371/journal.pone.0283817 (PMC10484457; doi:10.1371/journal.pone.0283817)
Supplement: S2 Fig — (PDF) [file pone.0283817.s002.pdf]

S2 Table: Partial data used for the construction of the graphs for the Figure 1 (A, B, and C).

Type	Sample Name	Temperature °C	Hydrodynamic diamenter d.nm	Polydispersity Index	Zeta potential mV
Size	CNE 1 D 1	25,0	195,0	0,225	
Size	CNE 1 D 2	25,0	197,7	0,248	
Size	CNE 1 D 3	24,9	193,5	0,277	
Zeta	CNE 1 D 1	25,0			-30,0
Zeta	CNE 1 D 2	25,0			-26,2
Zeta	CNE 1 D 3	25,0			-26,2
Size	ENE 1 D 1	25,0	119,1	0,279	
Size	ENE 1 D 2	25,0	117,4	0,297	
Size	ENE 1 D 3	25,0	117,8	0,281	
Zeta	ENE 1 D 1	25,0			-23,6
Zeta	ENE 1 D 2	25,0			-22,7
Zeta	ENE 1 D 3	25,0			-24,4
Size	CNE 15 D 1	25,0	195,0	0,274	
Size	CNE 15 D 2	25,0	189,7	0,272	
Size	CNE 15 D 3	25,0	191,6	0,281	
Zeta	CNE 15 D 1	25,0			-30,0
Zeta	CNE 15 D 2	25,0			-30,2
Zeta	CNE 15 D 3	25,0			-29,8
Size	ENE 15 D 1	25,0	118,1	0,290	
Size	ENE 15 D 2	25,1	118,4	0,305	
Size	ENE 15 D 3	25,0	117,7	0,276	
Zeta	ENE 15 D 1	25,0			-23,6
Zeta	ENE 15 D 2	25,0			-23,0
Zeta	ENE 15 D 3	25,0			-25,0
Size	CNE 30 D 1	25,0	186,3	0,262	
Size	CNE 30 D 2	25,0	186,6	0,262	
Size	CNE 30 D 3	25,0	185,9	0,278	
Zeta	CNE 30 D 1	25,0			-30,3
Zeta	CNE 30 D 2	25,0			-29,8
Zeta	CNE 30 D 3	25,0			-30,6
Size	ENE 30 D 1	25,0	115,1	0,287	
Size	ENE 30 D 2	25,0	116,2	0,295	
Size	ENE 30 D 3	25,0	114,1	0,266	
Zeta	ENE 30 D 1	25,0			-23,5
Zeta	ENE 30 D 2	25,0			-24,1
Zeta	ENE 30 D 3	25,0			-23,5
Size	CNE 60 D 1	25,0	185,9	0,255	
Size	CNE 60 D 2	25,0	183,9	0,252	
Size	CNE 60 D 3	25,1	183,5	0,263	
Zeta	CNE 60 D 1	25,0			-25,7
Zeta	CNE 60 D 2	25,0			-25,3
Zeta	CNE 60 D 3	24,9			-27,3

S2 Table: Partial data used for the construction of the graphs for the Figure 1 (A, B, and C).

(ENE 1 D 1 = Empty nanoemulsion, one day after synthesis, replicate 1 / CNE 1 D 1 = Copaiba nanoemulsion, one day after synthesis, replicate 1).
