# Supplementary material for: Synthesis of copaiba (Copaifera officinalis) oil nanoemulsion and the potential against Zika virus: An in vitro study
Source: PLoS One. 2023 Sep 7;18(9):e0283817. doi: 10.1371/journal.pone.0283817 (PMC10484457; doi:10.1371/journal.pone.0283817)
Supplement: S3 Fig — (PDF) [file pone.0283817.s003.pdf]

S3 Table: Data of the figure 3 (A) Cell viability after treatment with ENE.

Table format: **Grouped**		A			B			C		
		24 h			48 h			96 h		
		**A:Y1**	**A:Y2**	**A:Y3**	**B:Y1**	**B:Y2**	**B:Y3**	**C:Y1**	**C:Y2**	**C:Y3**
**1**	C	100.00	100	100	100.000000	100.000000	100.000000	100.00	100	100
**2**	5,6	103.51	83	123	106.400300	101.424400	95.434080	107.50	129	83
**3**	11,2	105.16	80	126	110.180400	100.540300	94.340840	121.08	140	200
**4**	22,5	114.65	81	145	119.759500	109.282900	106.430900	127.13	147	103
**5**	45	113.29	100	124	123.324700	112.917500	109.003200	162.77	122	200
**6**	90	109.43	87	127	113.573900	103.634600	98.392280	178.64	154	200
**7**	180	105.94	85	121	110.180400	109.332000	100.514500	209.16	155	259
**8**	360	118.74	90	144	116.237100	96.168960	101.414800	211.21	160	262
