# Supplementary material for: Synthesis of copaiba (Copaifera officinalis) oil nanoemulsion and the potential against Zika virus: An in vitro study
Source: PLoS One. 2023 Sep 7;18(9):e0283817. doi: 10.1371/journal.pone.0283817 (PMC10484457; doi:10.1371/journal.pone.0283817)
Supplement: S4 Fig — (PDF) [file pone.0283817.s004.pdf]

S4 Table: Data of the figure 3 (A) Cell viability after treatment with CNE.

Table format: **Grouped**		A			B			C		
		24 h			48 h			96 h		
		**A:Y1**	**A:Y2**	**A:Y3**	**B:Y1**	**B:Y2**	**B:Y3**	**C:Y1**	**C:Y2**	**C:Y3**
**1**	C	100.00	100.00	100.00	100.000000	100.000000	100.000000	100.00	100.0	100
**2**	5,6	109.10	107.00	107.00	101.676700	105.675700	99.016400	106.51	100.0	112
**3**	11,2	118.00	102.04	100.00	103.611400	101.621600	99.213110	117.97	110.0	120
**4**	22,5	99.11	98.00	98.00	111.693900	110.756800	105.704900	123.10	110.0	132
**5**	45	118.00	102.04	100.00	111.393000	104.756800	90.819670	148.10	160.0	132
**6**	90	100.00	112.00	102.49	98.739270	99.191890	93.630490	164.59	171.0	155
**7**	180	99.00	102.60	100.00	90.920030	91.837840	95.007320	180.00	160.0	143
**8**	360	98.00	98.84	101.00	76.356250	69.675670	84.127780	106.36	122.0	90
