# Supplementary material for: Synthesis of copaiba (Copaifera officinalis) oil nanoemulsion and the potential against Zika virus: An in vitro study
Source: PLoS One. 2023 Sep 7;18(9):e0283817. doi: 10.1371/journal.pone.0283817 (PMC10484457; doi:10.1371/journal.pone.0283817)
Supplement: S5 Fig — (PDF) [file pone.0283817.s005.pdf]

S5 Table: Data of the figure 3 (A) Cell viability after treatment with free copaiba oil.

Table format: **Grouped**		A			B			C		
		24 h			48 h			96 h		
		**A:Y1**	**A:Y2**	**A:Y3**	**B:Y1**	**B:Y2**	**B:Y3**	**C:Y1**	**C:Y2**	**C:Y3**
**1**	C	100.00	100	100	100.00	100	100	100.00	100	100
**2**	5,6	102.25	100	100	115.53	95	133	14.62	11	15
**3**	11,2	100.08	99	99	104.69	95	113	15.61	15	11
**4**	22,5	96.75	100	92	81.92	90	70	7.24	7	7
**5**	45	102.68	90	112	93.90	80	106	20.84	18	18
**6**	90	94.20	100	86	74.43	80	64	13.04	12	14
**7**	180	77.45	80	74	28.48	30	26	19.27	18	20
**8**	360	54.18	54	54	22.73	20	20	36.48	35	37
