# Supplementary material for: Synthesis of copaiba (Copaifera officinalis) oil nanoemulsion and the potential against Zika virus: An in vitro study
Source: PLoS One. 2023 Sep 7;18(9):e0283817. doi: 10.1371/journal.pone.0283817 (PMC10484457; doi:10.1371/journal.pone.0283817)
Supplement: S6 Fig — (PDF) [file pone.0283817.s006.pdf]

S6 Table: Data of the figure 5 (B) Relative foci number (%) resulting from the viral inhibition assay in VERO E6 cells infected with ZIKV.

First repeat

Treatment	nº focus	Reciprocal of dilution	Factor of dilution	TOTAL	Percentage
Control	30.5	0.5	100	1525	100
ENE	8.5	0.5	100	425	27.86885246
CNE	4.5	0.5	100	225	14.75409836
**Second repeat**					
Control	16	0.5	1000	8000	100
ENE	5	0.5	1000	2500	31.25
CNE	4	0.5	1000	2000	25

			Media	SD
**Control**	100	100	100	0
**ENE**	27.86885	31.25	29.55942623	2.390832
**CNE**	14.7541	25	19.87704918	7.244947
