# Supplementary material for: Synthesis of copaiba (Copaifera officinalis) oil nanoemulsion and the potential against Zika virus: An in vitro study
Source: PLoS One. 2023 Sep 7;18(9):e0283817. doi: 10.1371/journal.pone.0283817 (PMC10484457; doi:10.1371/journal.pone.0283817)
Supplement: S7 Fig — (PDF) [file pone.0283817.s007.pdf]

S5 Table: Data of the figure 3 (A) Cell viability after treatment with free copaiba oil.

Table format: **Grouped**		A									
		NCP									
		**A:Y1**	**A:Y2**	**A:Y3**	**A:Y4**	**A:Y5**	**A:Y6**	**A:Y7**	**A:Y8**	**A:Y9**	**A:Y10**
**1**	C	3.593200e+013	2.319680e+013	1.375120e+014	2.765320e+013	2.715090e+013	1.954510e+013	80757.66000	86747.18000	115701.50000	274727.900000
**2**	5,6	2.425950e+014	4.354340e+014	4.824530e+014	1.598520e+013	4.334120e+013	4.238620e+013	419035.80000	374419.90000	401534.10000	462335.000000
**3**	11,2	9.604912e+010	9.877166e+010	2.441690e+011	1.049830e+012	6.813020e+011	9.500610e+011	211617.10000	56280.05000	101823.20000	116782.800000
**4**	22,5	5.777570e+012	2.083900e+012	3.645450e+012	1.724260e+012	1.788660e+012	7.184111e+009	355972.70000	325026.70000	279716.30000	1.113424e+007
**5**	45	4.385500e+010	3.803639e+010	3.052859e+010	2.799822e+008	3.035124e+008	3.471085e+008	11509.68000	9641.26600	8538.14400	53244.140000
**6**	90	057235.000000	286728.000000	338169.000000	427886.000000	419570.000000	252723.000000	3174.38500	3284.52900	4603.72300	3025.557000
**7**	180	268526.000000	619683.500000	151860.500000	412593.000000	548505.000000	290926.000000	2339.95400	1833.27300	3152.66300	2577.956000
**8**	360	117466.000000	535015.000000	245843.000000	490188.000000	697531.800000	916126.200000	2777.86300	2519.15300	2684.01000	2751.238000

	A:Y11	A:Y12	A:Y13	A:Y14	A:Y15	A:Y16	A:Y17	A:Y18	B:Y1	B:Y2	B:Y3
1	447441.30000	435233.000000	88739.93000	94749.47000	102510.20000	5468.39000	4169.24300	8910.89800	8.59320e+013	2.31968e+013	1.37512e+014
2	1733600.00000	833881.000000	130.17200	220.67900	112.25100	7250.02700	7768.63400	6352.33300	173578.80000	242697.50000	218299.90000
3	278260.80000	191915.400000	9298.98400	8643.89900	9673.74500	1698.90100	1497.42000	1981.84400	367263.30000	402835.40000	540682.00000
4	9333802.00000	1.049681e+007	43645.33000	40926.79000	48642.48000	9609.71900	9828.02100	9822.48000	500456.40000	524524.00000	547006.00000
5	40170.45000	54384.950000	8110.74100	7644.32700	9309.84500	24102.16000	18557.51000	20769.07000	213352.20000	249777.90000	242219.60000
6	3676.05700	2202.950000	75.44400	52.89800	51.89100	3069.11900	2647.17400	3277.11700	461928.70000	530501.30000	240921.30000
7	2981.05600	2855.458000	601.80300	445.09200	434.77400	18361.29000	10848.14000	13077.01000	156233.60000	229484.60000	195911.50000
8	3027.63500	2491.339000	87.94000	81.74000	99.84300	900.53800	1465.06000	1310.36200	12656.56000	12179.00000	14256.71000

	B											
	NV											
	**B:Y4**	**B:Y5**	**B:Y6**	**B:Y7**	**B:Y8**	**B:Y9**	**B:Y10**	**B:Y11**	**B:Y12**	**B:Y13**	**B:Y14**	**B:Y15**
**1**	2.76532e+013	2.71509e+013	1.95451e+013	80757.660000	86747.180000	15701.500000	74727.900000	47441.300000	35233.000000	88739.93000	94749.47000	102510.20000
**2**	361231.50000	372842.80000	353160.10000	14432.660000	17303.810000	18577.540000	55707.520000	55873.820000	61911.130000	13549.61000	14725.53000	13615.55000
**3**	567508.60000	705739.80000	642253.90000	8695.784000	6936.187000	8123.200000	7061.042000	7380.113000	5743.891000	235108.10000	249878.10000	252055.90000
**4**	521028.40000	575340.30000	519277.90000	25638.670000	27245.840000	29732.360000	8247.683000	6709.061000	8758.153000	197486.00000	235315.80000	220280.20000
**5**	306193.10000	269716.70000	307835.80000	7597.167000	7711.806000	10633.900000	4359.924000	4799.686000	5306.235000			
**6**	603002.30000	522158.60000	333598.00000	41907.300000	22763.930000	36067.100000	5259.561000	3795.452000	5467.132000	154547.50000	152908.50000	148463.20000
**7**	272843.90000	246224.90000	198478.70000	61021.000000	24023.300000	22698.900000	37819.290000	33939.340000	39635.710000	16200.63000	17580.36000	15694.78000
**8**	54870.57000	87239.35000	77631.75000	58988.680000	75314.270000	82092.880000	7414.145000	8147.818000	9446.622000	2.66900	7469.11000	8299.99300

	B:Y16	B:Y17	B:Y18
**1**	5468.39000	4169.24300	8910.89800
**2**	23056.01000	26938.95000	37784.75000
**3**	94408.30000	81094.85000	87031.98000
**4**	21731.77000	22076.22000	21764.73000
**5**	25.20200	26.74700	29.55700
**6**	185281.60000	200614.80000	188812.30000
**7**	154019.80000	156020.80000	174682.70000
**8**	10960.01000		
